# Trends in clinical development timeframes for antiviral drugs launched in the UK, 1981–2014: a retrospective observational study

**DOI:** 10.1136/bmjopen-2015-009333

**Published:** 2015-11-16

**Authors:** Derek J Ward, Edward Hammond, Luan Linden-Phillips, Andrew J Stevens

**Affiliations:** 1 NIHR Horizon Scanning Research & Intelligence Centre, School of Health and Population Sciences, University of Birmingham, Birmingham, UK; 2 Department of Public Health, Epidemiology and Biostatistics, School of Health and Population Sciences, University of Birmingham, Birmingham, UK

**Keywords:** Viral infection, Drug development timeframes, Drug licensing, United Kingdom

## Abstract

**Objectives:**

Recent decades have witnessed the development of highly innovative new antiviral drug therapies. However, there are concerns that rising costs and lengthening development times could have implications for future patient access to innovative new drugs. We sought to establish whether the time taken for the clinical development of new antiviral drugs launched in the UK had increased since the 1980s.

**Design and setting:**

Retrospective observational study of all new antiviral drugs licensed for use in the UK.

**Primary and secondary outcome measures:**

Duration of clinical development (from initiation of studies in humans to receipt of Marketing Authorisation), subdivided into clinical trial and regulatory approval periods by the date of Marketing Authorisation Application.

**Results:**

48 new antiviral drugs were licensed for use in the UK between 1981 and 2014 (inclusive), over half (54%) initially for HIV infection. The overall mean duration of clinical development was 77.2 months, of which 64.6 months was spent in clinical trials before regulatory submission. The total time in clinical development increased from 41.7 months for drugs licensed 1981–1992 to 91.7 months for drugs licensed 2004–2014. This increase was accounted for by an increase in the clinical trials period and not the regulatory approval period, for which there was no observable trend. Drugs initially licensed to treat hepatitis C had a longer duration of clinical development than those indicated for other viral infections. However, the, initially shorter clinical development durations of drugs indicated for HIV infection increased more rapidly across the study period than those indicated for other viral infections.

**Conclusions:**

The time spent by antiviral drugs in clinical development has increased markedly in recent decades despite many initiatives to speed access to innovative new drugs. However, this represents only one part of the translational research pathway, and a complete picture of development timeframes is lacking.

**Strengths and limitations of this study**

- This is the most up to date and complete study that considers trends in clinical development timelines for new drugs introduced into the UK.
- The study used data from the European Medicines Agency, Medicines and Healthcare Products Regulatory Agency and British National Formulary to ensure that all relevant drugs were identified and regulatory dates were accurate.
- However, this study considered only the clinical phase of development, from the initiation of clinical trials to regulatory approval and omits the time and resources needed for discovery and preclinical development, as well as postauthorisation activities.
- This study did not consider new indications or the repurposing of existing licensed and marketed drugs.

## Introduction

Recent decades have seen the emergence and identification of several new viral infections of global significance,^1^ ^2^ but they have also witnessed the development of highly innovative new antiviral drug therapies, which have, for example, dramatically improved the prognosis of those infected with HIV^3^ and now are radically changing the care of those infected by hepatitis C.^4^ However, concerns have been expressed about the increasing costs of developing new drugs and bringing them into clinical use.^5–9^ In broad terms, drug development begins with discovery and preclinical laboratory research, before moving on to clinical development, starting with first testing in humans (phase I clinical trials) and continuing until regulatory review and approval.^5^ ^6^ ^10^ ^11^ Phase I to III clinical trials may be responsible for more than half the time and cost required to bring a new drug from discovery to regulatory approval.^6^ ^12^ Therefore, trends in the time taken for clinical development may be an important driver of increasing total development costs as well as having implications for patient access to innovative new medicines.

A number of authors have attempted to characterise the time taken to translate basic research findings into clinical practice; a review by Morris *et al*^10^ identified a number of studies reporting the time taken to translate health research, concluding that 17 years was the most likely estimate despite wide variation in definitions and marked differences in the time periods studied and approaches to data collection. However, few researchers have considered trends in drug development timeframes. Keyhani *et al*^13^ considered the time taken from the Investigational New Drug (IND) Application (a step required by the US Food and Drug Administration (FDA) prior to testing in humans^14^) to the filing of a New Drug Application^15^ and subsequent FDA approval (the latter time points representing the regulatory review process). For all new drugs approved in the USA from 1992 to 2001, the authors found no increase in the time taken to conduct clinical trials prior to filing a New Drug Application (median 5.1 years) and a decrease in the time taken for the subsequent regulatory review and approval. Kaitin and DiMasi^16^ considered a much longer time frame (1980–2009) and also found a decrease in time taken for regulatory review by the FDA. However, they found an increase in the clinical trial periods prior to filing (increasing from a mean 5.7 years for drugs approved in the USA 1980–1984, to 6.4 years for those approved 2005–2009) and concluded that this was due, in part, to increasing numbers of central nervous system and antineoplastic agents with very long average development times. However, the clinical trial periods prior to filing (ie, from IND application to filing a New Drug Application) for HIV antiviral agents also increased from a mean 2.3–5 years, and clinical trial periods for other anti-infective agents (including other antiviral drugs) increased from 4.2 to 6.6 years. More recent data on drug intervention trials registered with ClinicalTrials.gov suggests that trial lengths may have reduced in recent years (from 2 years for trials beginning in 2005 to 1.3 years for those beginning in 2009), but this trend was considerably less marked for industry sponsored trials, which are more likely to support the approval of new drugs.^17^

Similar data on trends in drug development have not been published for drugs launched in the UK. We sought to determine whether the overall time taken for the clinical development (clinical trial periods prior to filing and subsequent consideration by the regulator) of new drugs launched in the UK had changed over more than three decades, using data for antiviral drugs.

## Methods

All new drugs first licensed for use in the UK between 1981 and 2014 (inclusive) and specifically indicated for the treatment of viral disease were identified along with their initial approved indication(s) from relevant editions of the British National Formulary (BNF) and the European Medicines Agency (EMA) website. The BNF lists all preparations available for prescribing and/or dispensing in the UK, including prescription only and over-the-counter medicines. A new drug was defined as a new chemical entity or new biological product not previously licensed for use in the UK; new formulations and new indications for existing licensed drugs were omitted from the study, as were new combination products where all the active components were already licensed and available separately or in other combination products.

Clinical development was defined as the period from the initiation of studies in humans (clinical trials) to the receipt of a Marketing Authorisation (MA or ‘licence’) applicable to the UK from the Medicines and Healthcare Products Regulatory Agency (MHRA) or the EMA as appropriate.^5^ This period was further subdivided by the date of Marketing Authorisation Application (MAA, regulatory submission or ‘filing’) into periods representing clinical trials (prior to filing) and subsequent regulatory approval. The initiation of clinical development was determined from searches of a commercial pharmaceutical R&D database (Pharmaprojects, Informa Group plc) and a bibliographic database of published biomedical literature (MEDLINE, US National Library of Medicine). The date of IND Application to the FDA was taken as the start of clinical development; where this was not available, we used the date that the first clinical trials were undertaken (taken from the published literature) or the date that the first report of clinical trials (typically phase I) was published instead. The dates of MAA and MA were obtained from the EMA website or direct from the MHRA.

The duration of clinical development, as well as the clinical trial and regulatory approval periods, were calculated to the nearest month. Simple descriptive statistics were used to summarise these data and explore differences in durations according to the drug indication (viral disease) and year of licence (1981–1992, 1993–2003 and 2004–2014). The statistical significance of differences in mean duration were determined using unpaired t tests. Scatter plots were used to visualise trends in development timeframes by year of UK drug launch, and where relevant, Pearson's correlation coefficient and least-squares linear regression lines were calculated. Statistical analyses were conducted using SPSS (V.21.0, IBM).

## Results

There were 48 new drugs licensed for the treatment of viral diseases in the UK during the 34-year period from 1981 to 2014, representing a mean 1.4 new drugs per year (table 1). Almost half of these drugs were licensed in the middle period (1993–2003, 48%), while just 15% were licensed in the earlier period (1981–1992). Over half of new drugs (54%) were initially indicated for HIV infection. The next most frequent initial indication was hepatitis C infection (15%), followed by infection with cytomegalovirus (13%), hepatitis B (8%), herpes simplex virus (4%), influenza virus (4%) and respiratory syncytial virus (2%, full details provided in online supplementary file 1).

**Table 1:** Duration of clinical development for antiviral drugs, in total and subdivided into clinical trials and regulatory approval periods, by year of first license and indication in the UK, 1981–2014

	Number	Duration of development (months)								
		Clinical trials period			Regulatory approval period			Total duration		
		Mean	SE	95% CI	Mean	SE	95% CI	Mean	SE	95% CI
All new drugs	48	64.6	4.70	55.9 to 73.3	12.6	0.96	10.8 to 14.7	77.2	4.75	68.7 to 86.6
Drugs licensed between										
1981–1992	7	41.7	9.90	23.5 to 62.6	16.3	5.02	7.3 to 26.8	58.0	13.2	37.6 to 86.4
1993–2003	23	63.2	5.34	53.6 to 73.7	11.2	1.01	9.3 to 13.1	74.4	5.21	64.9 to 84.9
2004–2014	17	78.4	9.02	61.5 to 96.7	13.3	1.10	11.3 to 15.6	91.7	8.80	75.2 to 108.9
Initial licensed indication										
HIV infection	26	55.8	4.18	47.5 to 64.1	11.8	0.94	10.0 to 13.5	67.6	4.43	58.7 to 76.5
Hepatitis C infection	7	92.1	20.52	52.8 to 133.4	13.9	4.69	8.4 to 25.2	106.0	21.00	63.7 to 147.2
Other viral infection	15	66.8	7.97	51.2 to 83.3	13.5	1.59	10.3 to 16.4	80.3	7.32	66.6 to 95.3

**Figure 1:**
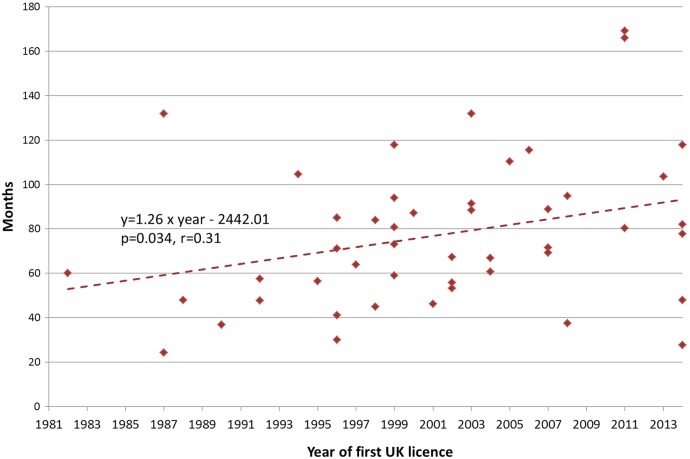
Duration of clinical development for antiviral drugs by year of first license in the UK, 1981–2014.

The overall mean duration of clinical development was 77.2 months, the majority of which was spent in clinical trials before the date of regulatory submission (84%, table 1). The total time in clinical development increased during the study period, from 58 months for drugs licensed 1981–1992, to 91.7 months for drugs licensed 2004–2014, a result that was statistically significant (p=0.048). This increase was accounted for by an increase in time spent in clinical trials before regulatory submission, which increased from 41.7 months for drugs licensed 1981–1992 to 78.4 months for drugs licensed 2001–2014 (p=0.027). No equivalent increase in time spent in the regulatory approval period was observed. A statistically significant upward linear trend in the total duration of clinical development (r=0.31, y=1.26×year—2442.01, p=0.034, figure 1) and the clinical trials period (r=0.34, y=1.38×year—2695.37, p=0.018) was observed for drugs licensed across the whole study period, suggesting that mean total clinical development and clinical trial durations increased by 12 months every 9.5 and 8.7-year period, respectively. In contrast, no significant linear trend was observed for the duration of the regulatory approval period (r=0.16, y=−0.12×year+253.36, p=0.32).

**Figure 2:**
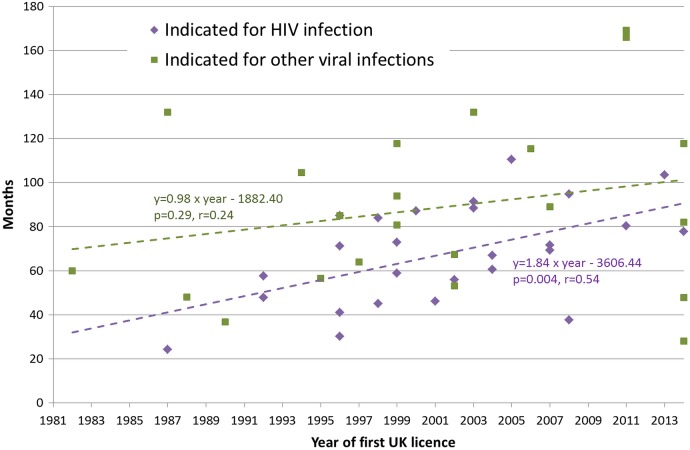
Duration of clinical development for antiviral drugs indicated for HIV and other viral infections by year of first license in the UK, 1981–2014.

Considering the initial indications for new drugs, those licensed to treat hepatitis C infection had a longer duration of clinical development than those indicated for HIV infection or other viral infections (table 1), though this result was not statistically significant. A statistically significant upward linear trend was observed for total clinical development durations for drugs first licensed to treat HIV across the study period (r=0.54, y=1.84×year—3606.44, figure 2). The clinical development durations of drugs initially licensed to treat other viral infections increased at a slower rate, and the trend was not statistically significant (r=0.29, y=0.98×year—1882.40).

## Discussion

We have shown that for drugs licensed in the UK to treat viral infections, the time spent in clinical development has increased markedly over the past three decades. This increase is due to increasing time spent in clinical trials before regulatory submission; no trend was observed in the time taken for regulatory approval. In addition, drugs licensed for hepatitis C appeared to spend longer in development than those for other viral infections but clinical development durations increased more rapidly for drugs licensed to treat HIV than those licensed to treat other viral infections. Our estimate of the rate of increase in mean time spent in clinical trials prior to regulatory submission is much greater than that found by Kaitin and DiMasi for all drugs approved in the USA between 1980 and 2009 (from 5.7 years in the period 1980–1989, to 6.4 years in the period 2000–2009).^16^ However, a more appropriate comparison might be between our results and those for anti-HIV drugs and other anti-infectives, where Kaitin and DiMasi reported a 117% and 57% increase, respectively, in the mean time spent in clinical trials between the periods 1980–1989 and 2000–2009. Our estimates of the mean time that antiviral drugs spent in clinical trials prior to filing increased 87% between the periods 1981–1992 and 2001–2014, and our estimated mean time spent in clinical trials for the period 2004–2014 (6.5 years for all antiviral drugs and 5.3 years for anti-HIV drugs) were similar to those reported by Kaitin and DiMasi for the period 2000–2009 (5 years for anti-HIV drugs and 6.6 years for other anti-infectives), showing a considerable degree of concurrence between their findings and our own.

This study relied on data from the BNF, MHRA and EMA, ensuring that the identification of drugs was comprehensive and the data on regulatory dates were accurate. For initiation of clinical trials, we relied on the date of IND application, a process which only applies to the USA and not to Europe. This point in the development of a new molecule occurs when the developer ‘wants to test its diagnostic or therapeutic potential in humans’.^14^ On a global basis, the IND application may be regarded as a reasonable proxy for the decision to advance development of a new drug beyond preclinical testing given that in recent decades the majority of new drugs have been launched in the USA at an earlier or similar time to Europe.^18^ The European Union (EU) equivalent process, requiring submission of a request for authorisation of clinical trials, stems from a 2001 Directive^19^ and is therefore a more recent development that could not be applied to the study period of interest. We could not determine a date for IND application in six cases (representing drugs launched between 1982 and 1996). The association between clinical development time and year of launch was maintained after excluding these drugs from the analysis (r=0.42, y=1.99×year—3912.46, p=0.006).

Our study addresses only the clinical development period of drug development; it omits the time and resources needed to discover and bring a candidate molecule through laboratory and initial animal studies, and does not consider activities postapproval, including marketing, phase IV studies, evidence reviews and the generation of guidance to clinicians and health services.^6^ ^11^ ^20^ Several commentators have described models of the translation pathway providing consistent definitions for different stages of the process and allowing comparison between studies and evaluation of efforts to reduce unwarranted delays. Trochim *et al*^11^ proposed a ‘process marker’ model that recognises translational research as a continuous process that may be ‘bidirectional, variable, (and) complex’ with observable milestones along a generalised pathway allowing measurement of time elapsed between them. Recognising that the process marker model may be more appropriate to technology driven developments, Hanney *et al*^20^ have proposed a generalisable model for all healthcare innovation, suggesting a matrix of four different ‘tracks’ in the innovation process, building from discovery research, through human research and research review, to clinical and health service/public policy development and finally clinical practice. This recognises the overlapping nature of research translation activities, and allows for the initiation of different tracks at different times despite relevant research still occurring in ‘earlier’ tracks. Our study considers the ‘Clinical Trials System’ element of the translational research continuum described by Trochim *et al*^11^ and overlapping elements of tracks 2 and 3 in Hanney *et al*'s^20^ description of the innovation process. Taken together, these help identify drivers of the trends seen. In this way, the increasing demands of regulators that have led to increasing complexity of clinical trial programmes are highly relevant to the increase in clinical trial periods we have observed. This has been recognised by policymakers, who have agreed revisions to the 2001 EU Clinical Trials Directive (effective from 2004), widely seen as having a negative impact on translational research, increasing administrative burdens and waiting periods^21–24^ and introduced a range of initiatives in the USA and Europe to provide a more rapid pathway to approval for innovative drugs that address serious conditions, urgent public health needs or specific patient groups.^25^ ^26^

Despite the introduction of these initiatives, visual inspection of our data (figures 1 and 2) shows no apparent step change consistent with a sudden change in the regulatory environment (such as the creation of the EMA or implementation of the EU Clinical Trials Directive). This may be expected given the long lead times for planning and conducting clinical trial programmes, though further analysis shows no association between the duration of clinical development and the year when clinical development started (r=0.04, y=−0.16+399.67, p=0.80). However, the impact of regulators can be seen in the shorter development durations seen for HIV drugs, which have previously been reported as receiving Marketing Authorisation without ‘large-scale human clinical trials’,^27^ though our data suggest this difference is now lessening. This is an example of regulators responding to a public health need and being willing to adopt a different view of the risk-benefit in specific circumstances.^20^ One current regulatory development applicable to the UK is the MHRA's Early Access to Medicines Scheme, which allows patients with serious conditions to access medicines that have not yet been approved where there is a clear unmet medical need.^28^ This will not necessarily reduce time to Marketing Authorisation, but may facilitate collection of real-world data to support earlier adoption of innovative new drugs. In this way it has parallels with the EMA's adaptive licensing pilot, but this EU-wide initiative also has the potential to bring forward the date of a drugs initial Marketing Authorisation, allowing first approval in highly selected patient populations based on more limited initial clinical studies.^29^ Both these examples are indicative of a wider move towards early dialogue between regulators and commercial developers,^26^ a move mirrored by health technology assessment (HTA) agencies in Europe, either alone or in parallel with regulators.^26^ ^30^ Early dialogue may or may not lead to a reduction in clinical development durations, but it is likely to be reflected in the design and conduct of late-phase clinical trial programmes.^30^ ^31^ This will have an uncertain effect on clinical trial times if HTA agencies demand longer term patient-related outcomes rather than proxy measures on which to judge value, but it may be expected to speed up the total time to adoption and diffusion of those drugs judged to be most clinically and cost-effective.

The past few decades have seen the introduction of many novel antiviral drugs, but the time spent in clinical development before drug approval has increased substantially. We found no evidence that this trend in increasing clinical development timescales is levelling off, though many of the current initiatives aimed to speed access to innovative new medicines are too recent to have affected clinical trial programmes for drugs launched up to 2014, and will therefore require further evaluation in the coming years. However, it is important to stress that adequate time spent in clinical trials is critical to generate evidence of safety, efficacy and cost-effectiveness^20^ and that regulators require adequate time to consider this evidence and strike an appropriate balance between benefit and risk.^32^ There are necessarily limits to clinical development durations, but this is only one part of the whole discovery to clinical practice translational pathway and increased time in one part of the pathway may be offset by gains elsewhere. Further research should incorporate measures of preclinical and adoption timeframes in order to gain a complete picture and ensure there are no unwarranted delays in novel drugs reaching patients in need.
